# Molecular detection and dense granule antigen 6 genotyping of feline *Toxoplasma gondii* in Phayao, Thailand

**DOI:** 10.14202/vetworld.2022.2309-2314

**Published:** 2022-09-26

**Authors:** Chittakun Suwancharoen, Chorpaka Phuangsri, Khanuengnij Prakhammin, Ornampai Japa

**Affiliations:** 1 Division of Microbiology and Parasitology, School of Medical Sciences, University of Phayao, Phayao, Thailand; 2 Department of Applied Statistics, Rajamangala University of Technology Isan, Khon Kaen Campus, Khon Kaen, Thailand; 3 Scientific Instrument and Product Standard Quality Inspection Center, University of Phayao, Phayao, Thailand

**Keywords:** domestic cats, genotype, granule antigen 6, *Toxoplasma gondii*, toxoplasmosis

## Abstract

**Background and Aim:**

Globally, toxoplasmosis is an important zoonotic parasite infection of many warm-blooded animals (including humans). *Toxoplasma gondii* oocysts are widespread, and their contamination can be primarily attributed to the members of the Felidae family. This study aimed to estimate the prevalence and determine the dense granule antigen 6 (GRA6) genotype of *T. gondii* among domestic cats in the Phayao Province, Thailand.

**Materials and Methods:**

A total of 124 fecal samples were collected from owned cats in the Muang district, Phayao, Thailand, from January to December 2020. Fecal samples were tested for the presence of *T. gondii* DNA using targeted B1 gene polymerase chain reaction (PCR) amplification, and positive samples were subsequently analyzed for their *T. gondii* genotype through PCR-restriction fragment length polymorphism (RFLP) analysis and sequencing of the GRA6 gene.

**Results:**

Among the 124 samples, 46 (37.1%) were tested positive for *T. gondii*. Only 10 positive DNA samples were successfully amplified for the GRA6 marker. Subsequent PCR-RFLP and sequence analyses indicated that all *T. gondii* isolates from cats in Phayao belonged to GRA6 genotype I.

**Conclusion:**

Data revealed that toxoplasmosis is remarkably distributed among (studied) domestic cats in Phayao, Thailand. Moreover, the virulent GRA6 allele was found to be circulated among domestic cats in this area. However, no significant correlation was observed between infection rates and different risk factors, which indicated that pet cats of any age, gender, or breed have similar risks of being infected with *T. gondii*. Our results further suggested that infective oocysts of *T. gondii* are widely distributed and that environmental contamination with these oocysts will introduce more risks of disease transmission to humans and other animals.

## Introduction

In recent years, pet populations have continuously increased in Thailand and other countries [1–3]. This close contact between humans and domestic animals creates favorable conditions for the transmittance of several zoonotic infectious diseases [4]. Toxoplasmosis, caused by the tissue cyst-forming coccidian *Toxoplasma gondii*, is a common zoonotic parasite infection associated with pet cat ownership [5]. In addition, *T. gondii* is considered as an important zoonotic agent that can infect most warm-blooded animals (including humans) [6, 7]. Although toxoplasmosis is generally asymptomatic or mild, it may cause serious infections or even death in human or animal hosts, particularly immunocompromised hosts, including aged, pregnant, or young as well as those suffering from malnutrition [8, 9]. Furthermore, disease severity varies depending on the genotype of *T. gondii* strains [10, 11]. Genetically, the latter can be classified into three major strain types (genotype or types I, II, and III) that differ in virulence and pathogenicity [12]. Among these strain types, *T. gondii* genotype I is considered as the most virulent strain.

Many vertebrate animals are involved in the life cycle of *T. gondii*. For example, a wide range of warm-blooded animals serves as intermediate hosts, whereas cats and other felids serve as the main definitive host of this parasite. Infected domestic cats significantly contribute to the spread of *Toxoplasma* oocysts throughout the environment [13, 14]. Thus, these felines play an important role in maintaining the sexual reproduction phase of *T. gondii* life cycle by producing a high number of oocysts in their feces.

Cats could be the main source of toxoplasmosis outbreaks; thus, accurately assessing the occurrence of *T. gondii* oocysts excreted by domestic cats is important. Moreover, understanding the prevalence of *T. gondii* among cat populations in the community is essential for effective surveillance, prevention, and control of toxoplasmosis.

Although the detection of *T. gondii* oocysts in cat fecal matter is conventionally achieved through microscopic examination, this method does not provide information regarding the virulent potential of detected *T. gondii* strains. Therefore, molecular detection of parasite DNA could be used for screening and typing of *T. gondii*. Given the absence of epidemiological data regarding feline toxoplasmosis and genotyping in North Thailand, this study aimed to estimate the prevalence and determine the dense granule antigen 6 (GRA6) genotype of *T. gondii* in domestic cats in Phayao Province.

## Materials and Methods

### Ethical approval

The study was approved by Animal Ethics Committee, University of Phayao (Approval number: 63 02 04 002).

### Study period and location

This study was conducted from January to December 2020. Fecal samples were obtained from cat litter boxes of 124 domestic cats reared in Muang district, Phayao Province (19°11′31″N 99°52′43″E), Thailand. All laboratory examinations were performed at the School of Medical Sciences and the Scientific Instrument and Product Standard Quality Inspection Center, University of Phayao, Phayao, Thailand.

### Study design and population

This work was a cross-sectional descriptive study. Only apparently healthy cats were randomly selected for this study. All cats were considered as owned cats. Basic information regarding age (months), sex (male/female), and breed (local/exotic/crossbred) were obtained from their owners. Cats missing any data were excluded from the study.

### Fecal DNA extraction

*Toxoplasma gondii* oocysts were separated from fecal samples (2 g) in accordance with the flotation method of ZnSO_4_ solution [15]. Flotation materials were harvested and washed 3 times with phosphate-buffered saline, after which DNA was extracted using the QIAamp Stool Mini Kit (following the manufacturer’s protocols). DNA samples were stored at −20°C for subsequent molecular detection and genotyping.

### Polymerase chain reaction (PCR) detection of *T. gondii*

The presence of *T. gondii* DNA was detected by targeting 115 bp of *T. gondii* B1 gene using *T. gondii* species-specific primers (B22: 5′-AACGGGCGAGTAGCACCTGAGGAGA-3′ and B23: 5′-TGGGTCTACGTCGATGGCATGACAAC-3′) [16, 17]. The PCR reaction volume (25 mL) included 5 pmol of each primer and 1 mL of cat fecal DNA. The PCR amplification was performed as follows: 94°C for 5 min, followed by 40 cycles of 94°C for 30 s, 60°C for 30 s, and 72°C for 1 min, and a final extension at 72°C for 5 min. DNA of *T. gondii* was included as a positive control, whereas DNA of related protozoa (i.e., *Neospora caninum*, *Giardia lamblia*, and *Sarcocystis* spp.) and distilled water were included as negative controls. The PCR products were separated and viewed using 2% agarose gel electrophoresis (Nancy-520 pre-stained).

### Granule antigen 6 (GRA6) genotyping of *T. gondii*

All B1 PCR-positive samples were detected by GRA6-nested PCR. A partial fragment of the GRA6 gene was amplified using two specific primer sets: GRA6F1 (5′-ATTTGTGTTTCCGAGCAGGT-3′) and GRA6R1 (5′-GCACCTTCGCTTGTGGTT-3′) for the first PCR and GRA6F2 (5′-TTTCCGAGCAGGTGACCT-3′) and GRA6R2 (5′-TCGCCGAAGAGTTGACATAG-3′) for nested PCR [18].

Each PCR reaction (25 μL) included GoTaq Flexi DNA Polymerase (Promega, Madison, WI, USA), 5 pmol of each primer, and 1 μL of DNA sample. The first PCR round was conducted under the following conditions: 94°C pre-denaturation for 5 min; 35 cycles of 94°C for 30 s, 55°C for 30 s, and 72°C for 1 min, followed by a final extension at 72°C for 5 min. The nested PCR (with nested primers) was performed using 1 μL of the first round PCR product. The same PCR setup as the first round was used, and a similar program was followed for 40 cycles, except for high-temperature annealing at 58°C. Amplified PCR fragments were analyzed using 1.5% agarose gel electrophoresis.

A total of 344 bp GRA6 PCR products were excised from agarose gel, purified, and subsequently digested in a 20 μL reaction mixture using the restriction enzyme *MseI*, which were incubated at 65°C for 16 h. The PCR-restriction fragment length polymorphism (RFLP) patterns were visualized using 2% agarose gel electrophoresis. In addition, the PCR product was cloned into a pGEMT vector before DNA sequencing was conducted by Macrogen, Korea.

### Sequence analysis

DNA sequences of GRA6 PCR products were assembled and processed using BioEdit version 7.2.5 (Ibis Biosciences, Carlsbad, CA, USA) [19]. Online sequence similarity searches were conducted using blastn (http://blast.ncbi.nlm.nih.gov/) and ToxoDB (https://toxodb.org/toxo/app/workspace/blast/new). In addition, GRA6 sequences were analyzed (*in silico*) for *MseI* restriction enzyme digestion using NEBcutter V2.0 (New England Biolabs Inc., USA) at http://nc2.neb.com/NEBcutter2/[20]. *T. gondii* GRA6 nucleotide sequences obtained in the present study were deposited into the GenBank database (accession number: ON665006-ON665015). Moreover, the phylogenetic tree was built using the maximum-likelihood method in the IQ-tree web server at http://iqtree.cibiv.univie.ac.at/ [21]. The GRA14 sequence of *N. caninum* [KM241868.1] was included as an outgroup. The final tree was visualized by FigTree 1.4.4 (The University of Edinburgh, Edinburgh, Scotland).

### Statistical analysis

The prevalence of *T. gondii* infection was determined as the proportion of infected cats to the total number of examined cats. A Chi-square test was performed to assess the correlation between the prevalence of infection and variable risk factors. Differences were considered to be statistically significant when p ≤ 0.05.

## Results

### Domestic cats studied

Of the 124 cats examined, 62 were female and 62 were male. Cats were aged between 2 months and 4 years (with an average age of 11.8 months). They were further allocated into two age groups: <1 year (83) and ≥1 year (41). Based on cat breeds, there were 45 local (Thais), 42 exotic (Persians, Scottish Fold, American Shorthairs, and Himalayan), and 37 crossbred.

### Prevalence of *T. gondii*

**Figure-1:**
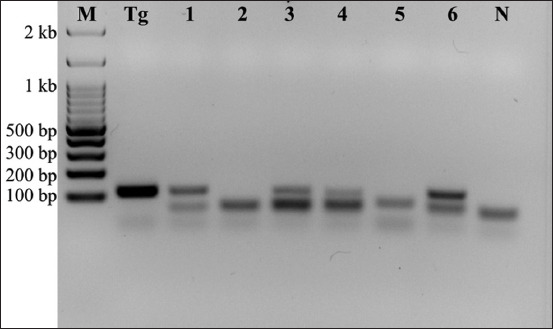
Representative B1 polymerase chain reaction products indicate the detection of *Toxoplasma gondii* from domestic cats in Phayao, Thailand. Lane M: 100 bp DNA ladder; lane Tg: Positive control (reference strain *T. gondii*); lanes 1, 3–4, and 6: Positive *T. gondii* samples; lanes 2 and 5: Negative samples; lane N: Negative control (*Neospora caninum* DNA).

DNA examination indicated a high prevalence of *T. gondii* infection among the 124 cat fecal samples. A 115 bp PCR product was amplified for 46 fecal samples (Figure-1). Positive samples were randomly selected to confirm the presence of *T. gondii* through B1 PCR product sequencing.

The overall rate of *T. gondii* infection was 37.1% (46/124). In addition, the prevalence of toxoplasmosis was slightly higher (41.0%) in young cats aged under 1 year than in older cats (29.3%) aged over 1 year. Up to 43.5% of female cats were found to be positive for *T. gondii* infection, whereas the latter only reached 30.7% in male cats. Regarding cat breeds, a higher percentage of infection was recorded for local (44.4%) followed by crossbred (35.1%) and exotics (31.0%). Regarding *T. gondii* infection, no significant association could be found among age groups, genders, or cat breeds. The prevalence of *T. gondii* infection based on each parameter is summarized in Table-1.

**Table-1:** Prevalence of *Toxoplasma gondii* infection in domestic cats from Muang District, Phayao, Thailand.

Parameters	No. of examined	No. of positive	No. of negative	Prevalence (%)	Chi-square	p-value
Age					1.609	0.205
< 1 year	83	34	49	41.0		
≥ 1 year	41	12	29	29.3		
Sex					0.137	2.212
Male	62	19	43	30.7		
Female	62	27	35	43.5		
Breed					1.782	0.41
Local	45	20	25	44.4		
Exotic	42	13	29	31.0		
Crossbred	37	13	24	35.1		
Total	124	46	78	37.1		

### Genotyping of *T. gondii*

**Figure-2:**
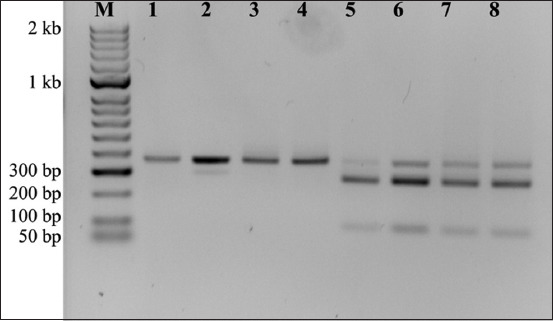
Polymerase chain reaction (PCR)-restriction fragment length polymorphism patterns for granule antigen 6 (GRA6) *Toxoplasma gondii* PCR products from domestic cats in Phayao, Thailand. Lane M: DNA markers; lanes 1–4: Undigested PCR product of GRA6 positive samples; lanes 5–8: *MseI*-digested GRA6 with a type I *T. gondii* genotype.

All PCR-positive samples (n = 46) were subsequently analyzed using GRA6 genotyping. Amplified fragments with an expected size of 344 bp were obtained through nested PCR amplification. Only 10 samples successfully generated GRA6-RFLP profiles. All samples exhibited a type-I genotype-specific pattern displaying two bands of 258 and 86 bp (Figure-2). No unusual or atypical genotypes were detected in the GRA6 locus.

### Granule antigen 6 sequence analyses

Sequence analysis of GRA6-positive samples (with 98.55–100% sequence homology) was performed on a 344 bp amplicon. For all isolates, the obtained *T. gondii* sequences showed great similarity to 344 bp GRA6 gene region sequences of *T. gondii*, which were available on GenBank and ToxoDB databases. Among the 10 GRA6-positive samples, three sequences were identical. Such sequences exhibited the highest similarity (with 100% homology) to that of *T. gondii* RH-88. Sequence and blast analyses of other positive samples further revealed 99.13–99.71% homology to *T. gondii* RH-88 (genotype I) compared with the 98.26–99.13% homology to *T. gondii* ME49 (genotype II) and *T. gondii* VEG (genotype III).

Analysis of *MseI* sequence digestion revealed one *MseI* recognition site (TTAA) at nucleotide position 258/259 within the 344 bp *T. gondii* GRA6 sequence, which resulted in two fragments (258 and 86 bp). The *MseI*-RFLP profile for *T. gondii* GRA6 sequences was consistent with the results obtained in the PCR-RFLP assay.

**Figure-3:**
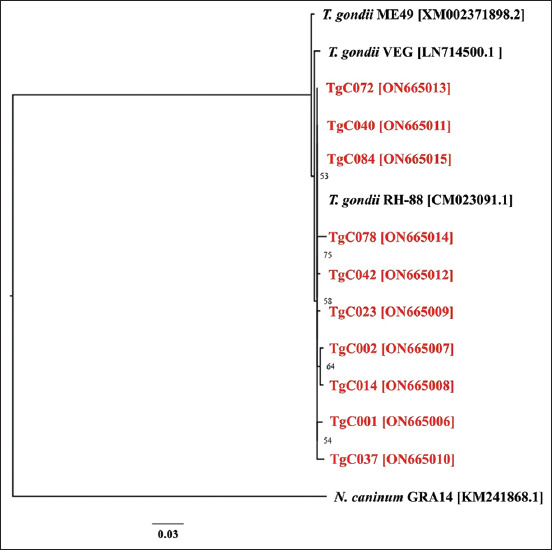
Phylogenetic relationships between 10 new *Toxoplasma gondii* isolates from domestic cats in Phayao, Thailand, and *T. gondii* type I-III reference strains based on their granule antigen 6 sequences. The phylogenetic tree was generated with 1000 bootstrap replicates using PhyML. Only bootstrap values of 50% or more are indicated at the nodes. Sequences from the present study are labeled in red letters.

Phylogenetic analysis was performed to assess the relationship between new *T. gondii* isolates and each clonal lineage based on their representatives (Figure-3). The new *T. gondii* isolates from Phayao cats clustered within *T. gondii* genotype I. Thus, phylogenetic analysis confirmed the findings of RFLP and blastn analyses.

## Discussion

The present study revealed a high prevalence of oocyst shedding in cats, in which 37.1% (46/124) of Phayao cats have *Toxoplasma* DNA in their feces, which might be *T. gondii* DNA stemming from oocysts. This prevalence was higher than that previously reported in Thailand (4.7%) [22] and neighboring countries such as Malaysia (8.5%) [23] and Indonesia (33.3%) [24]. By contrast, Norhamizah *et al*. [25] recorded a high incidence of toxoplasmosis; 40.98% of *T. gondii* oocyst was found in cat feces in Malaysia using modified Kato–Katz and Sheather’s sugar methods. *Toxoplasma* oocysts were identified by morphological characteristics, but the researchers did not include the molecular identification of *T. gondii*.

Microscopic examination of fecal oocysts is a low-cost and rapid method for direct examination. It also has limited sensitivity. This method also requires laboratory experience in identifying parasite oocysts as *T. gondii* oocysts are similar, which are often misdiagnosed with another coccidian [26]. Thus, the prevalence of toxoplasmosis among regions may vary because of geographical variations and selected specimens and methods for parasite detection [27]. In the present study, oocysts in cat feces were initially separated from fecal materials based on the density through flotation before conducting PCR detection. These processes could increase the potential of detecting *T. gondii* DNA.

In general, *T. gondii* oocysts are not frequently found during routine stool examinations with only 1% of infected cats reportedly excreting oocysts in a given period [14, 28]. Based on our findings, the high rate of *T. gondii*-infected cats in this area could contribute to oocyst burden in the environment, although infected cats only excrete oocysts early on after infection [27, 29]. Nevertheless, several oocysts can still be produced daily for 3 weeks after infection [29, 30], and they can survive for several months or even years [31]. These contaminated oocysts can be infectious to humans and other animals, which may cause public health problems [3, 32–34]. Moreover, infected felids in a high *Toxoplasma* prevalence area can be exposed to oocysts and may be reinfected, resulting in the re-excretion of more oocysts.

In the present study of *T. gondii* oocyst shedding among cats, the lack of statistical significance regarding age groups, breeds, and gender indicated that these factors were insignificant factors for *T. gondii* infections in cats. In addition, the role of age in the epidemiology of feline toxoplasmosis among cats in many regions has been determined, showing that the seroprevalence of *T. gondii* increased with age [35–37]. However, other studies have shown that age had no significant effect on the prevalence of *T. gondii* oocyst excretion in cats [38, 39]. Thus, the findings of the present study indicated that cats of any age can excrete *T. gondii* oocysts.

However, the effects of domestic feline breeds or gender on *T. gondii* oocyst excretion are unclear [40]. Although the current results revealed a higher proportion of oocyst shedding among locally bred cats (44.4%) over exotic (31.0%) or crossbred cats (35.5%), this difference was not significant (p > 0.05). Nevertheless, this result was consistent with the findings of the previous studies [38]. With regard to gender affecting the prevalence of *T. gondii* oocyst shedding, most previous reports suggested no effect, which was consistent with the current findings [39]. By contrast, Nabi *et al*. [38] reported a high proportion of infection among male cats.

Several genetic markers (e.g., rhoptry proteins [ROP5,16,18], surface antigen genes [SAG2,3], and dense granule antigens [GRA1-15]) have been utilized for *T. gondii* strain typing [41]. Among these markers, GRA genes are generally used for genetic characterization and typing of *T. gondii* isolated from humans, animals, and meat products [42–44]. The GRA6 gene is a polymorphic single-copy gene that may be involved in the pathogenicity and antigenicity of *T. gondii* [45]. The present study revealed the circulation of the type-I GRA6 allele in fecal samples of domestic cats in Phayao, Thailand. This result was consistent with the detection of type-I *T. gondii* GRA6 in domestic cats in South Thailand [46] and Okinawa, Japan [47]. Other studies have considered type-II *T. gondii* as the predominant genotype for feline definitive hosts in China [44] and stray cats in Iran [48]. Furthermore, isolates of *T. gondii* types I and II have been detected via GRA6 marker PCR-RFLPs in oocyst-contaminated soil samples from North Iran [49].

## Conclusion

The present study demonstrated a high occurrence of *T. gondii* DNA in fecal samples obtained from pet cats in Phayao, Thailand. Analyses of PCR-RFLP patterns and nucleotide sequences confirmed the presence of type-I *T. gondii*. These results indicated the circulation of type-I *T. gondii* GRA6 in this area, although such results were based on a single locus. However, data on circulating *T. gondii* genotypes from different hosts in Thailand are limited; thus, further investigations are necessary. In particular, epidemiological data of other animal species related to the life cycle of *T. gondii* and analyses of population genetics were required to accurately identify the circulating genotype of *T. gondii* in Thailand and comprehensively understand the relationship of toxoplasmosis in humans and animals.

## Authors’ Contributions

OJ, CS, and KP: Conceived and designed the study. OJ and CP: Collected the samples. OJ, CS, and CP: Performed the experiments. OJ: Conducted molecular work and performed sequence analyses. OJ and KP: Analyzed the data. OJ and CS: Drafted and revised the manuscript. All authors have read and approved the final manuscript.
